# Epithelial ovarian cancer subtypes attributable to smoking in the Norwegian Women and Cancer Study, 2012

**DOI:** 10.1002/cam4.590

**Published:** 2016-01-14

**Authors:** Idlir Licaj, Marko Lukic, Mie Jareid, Eiliv Lund, Tonje Braaten, Inger Torhild Gram

**Affiliations:** ^1^ Department of Community MedicineFaculty of Health SciencesThe UiT Arctic University of NorwayTromsøNorway

**Keywords:** Epithelial ovarian cancer, cohort study, histological subtypes, invasiveness, population attributable fraction, smoking

## Abstract

Among European women, ovarian cancer is the fifth most common cancer. Smoking is an established risk factor for mucinous tumors. We estimated the impact of smoking in Norwegian women using population attributable fractions (PAFs) of epithelial ovarian cancer (EOC), by invasiveness and by histological subtypes in the Norwegian Women and Cancer Study with an average of 13.2 years of follow‐up. During >2 million person‐years, a total of 915 incident EOC cases, of which 667 (73%) invasive and 248 (27%) borderline, were identified among 154,234 women aged 34–70 years at enrolment. Compared with never smokers, current smokers had a nonstatistically significant increased risk of mucinous tumors (hazard ratio [HR] = 1.67 [95% confidence interval, (CI), 0.96–2.96]) and more than twice statistically significant risk of borderline mucinous tumors (HR = 2.17 [95% CI, 1.06–4.45]). The corresponding PAF estimates were 16.5% for mucinous and 25% for borderline mucinous. We found that among middle‐aged women, one in six mucinous tumors and one in four borderline mucinous tumors could have been prevented if women did not smoke.

## Introduction

Among European women, ovarian cancer is the fifth most common cancer with more than 70,000 new cases recorded in 2012 1. Recently, a large European population study estimated that almost one in five new cancers are caused by cigarette smoking 2 while another report from the United Kingdom estimated that 2.6% of overall ovarian cancer cases are attributable to smoking 3. In 2012, the International Agency for Research on Cancer classified mucinous ovarian tumors as causally related to tobacco smoking based on results from twenty different epidemiological studies 4.

The Norwegian Women and Cancer Study (NOWAC) is a nationally representative prospective cohort providing a unique setting to estimate both the prevalence of smoking and the relationship of smoking and subtypes of epithelial ovarian cancer. We aimed to estimate the impact of smoking on epithelial ovarian cancers (EOC) using population attributable fractions (PAFs) of subtypes of EOC, by invasiveness status EOC and by histological subtypes in the NOWAC study.

## Material and Methods

### NOWAC study

The NOWAC is a national representative prospective cohort study initiated in 1991. The cohort has been described elsewhere 5, 6. Briefly, the Central Population Register selected a random sample of women according to year of birth. Subsequently, an invitation to participate in the study, with a baseline questionnaire and prestamped return envelope enclosed was mailed to these women. The NOWAC study was approved by the Regional Committee for Medical Research Ethics and the Norwegian Data Inspectorate. All women gave written informed consent (http://site.uit.no/nowac).

Women aged 34–70 years who completed a baseline questionnaire on lifestyle factors during three waves of data collection: 1991–1992, 1996–1997, and 2003–2007 (*N* = 172,539) were included. New women were recruited in each wave. We excluded women with prevalent cancer at baseline (except nonmelanoma skin cancer) (*N* = 6964), who emigrated or died before start of follow‐up (*N* = 80), with bilateral oophorectomy (*N* = 5760), who were born after 1957 (*N* = 3167) since they did not receive complete questionnaires, who emigrated after enrolment, but were diagnosed with cancer afterward (*N* = 13) and those with missing information on smoking status (*N* = 2260). The analytical cohort comprised the remaining women (*N* = 154,234).

The women reported if they have smoked, number of cigarettes smoked daily at different ages, and if they currently smoked daily. Women who had never smoked and reported no exposure to smoking in their childhood home were categorized as never smokers; those passively exposed to smoke during childhood were classified as passive smokers. We did not have complete information on passive smoking in adulthood. We categorized the women as current, former, passive, and never smokers based on this information.

Information on cancer incidence, emigration and deaths were obtained through linkages to the Cancer Registry of Norway (http://www.kreftregisteret.no/en) and Norwegian Central Population Register, respectively. The overall estimated completeness of the Cancer Registry of Norway has been shown to be more than 98% 7.

Person‐years were calculated from start of follow‐up until the date of any incident cancer diagnosis (except nonmelanoma skin cancer), emigration, death, or the end of follow‐up (31 December 2012), whichever occurred first. Epithelial ovarian cancer cases were classified using the International Classification of Diseases, ICD‐7 (location 175) and ICD‐O‐3 (tumor subtype) codes. Invasive and borderline surface epithelial–stromal ovarian tumors are here referred to as EOC.

### Statistical analysis

Cox proportional hazards models with age as the underlying time scale were used to estimate multivariable‐adjusted HRs with 95% confidence intervals (CI) for the associations between smoking exposure (passive, former, current) and EOC stratified by invasiveness and histological subtypes (serous, mucinous). Similar models were used to estimate multivariable‐adjusted HRs with 95% CI for the associations between different measures of smoking exposure (passive, former, current); and for current smokers; (age at smoking initiation [<20, 20+ years], smoking duration [≤20, 20+ years], number of pack‐years [<10, 10+], number of cigarettes smoked per day [<10, 10+]), and EOC overall and stratified by invasiveness and histological subtypes. We analyzed serous, mucinous and, endometrioid subtypes as separate groups and the remaining tumors, including clear cell, as “others”; we did not have enough cases of clear cell subtype to analyze this as a separate group. Never smokers were used as reference group.

Each of the following factors was found to be a potential confounder and included in the final model: age at menarche (≤12, >12 years), number of full‐term pregnancies (0, 1 or 2, 3+), age at first full‐term pregnancy (nulliparous, ≤19, >19–24, >24–29, and ≥30 years), age at last birth (≤24, >24–29, >29–33, and ≥34 years), infertility (yes, no, missing [*n* = 9625]) menopausal status (pre‐ or perimenopausal, postmenopausal, hysterectomy before 53 years, hormonal replacement therapy use before 53, missing [*n* = 3723]), age at menopause (≤45, >45‐50, >50–52, ≥53 years), educational attainment (≤9, >9–12, >12–16, ≥17 years, missing [7915]), physical activity score (scored as 1–5 low to high level, missing [*n* = 12,644]), alcohol intake (teetotalers, ≤4, >5–9, and, ≥10 g/day, missing [*n* = 8058]), BMI (≤18.49, >18.49–24.9, >24.9–29.9, ≥30 kg/m^2^, and missing [*n* = 3594]), oral contraceptive use (yes, no, missing [*n* = 5182]), duration of oral contraceptive use (≤3, >3–7, ≥8 years), hormonal replacement therapy (yes, no, missing [*n* = 30,864]), age at start using hormonal replacement therapy (≤45, >45–49, ≥50 years). We included missing indicators specific to confounding factors after checking that the parameters associated with these indicators were not associated with the different outcomes.

An ordinal exposure variable with equally spaced scores, which included never smokers, was created to test for linear trends. Wald chi‐square statistics were used to test for heterogeneity between different histological tumors types and according to the invasiveness status. Models were stratified by enrolment waves in order to control for differences in questionnaire design and follow‐up time. Schoenfeld residuals were used to test the proportional hazards assumptions. Effect modification in the relation between smoking status (never, passive, former and current) and EOC by, in turn, alcohol consumption (continuous), educational attainment, BMI and menopausal status (yes, no), and number of children was assessed. Models with main effects and interaction terms were fitted and compared with models with main effects only. The difference in log‐likelihood (likelihood ratio test statistics) was compared to a *χ* ^2^ distribution with degrees of freedom equal to the number of interaction terms.

We calculated the PAF in order to estimate the proportion of EOC subtypes that would not occur if smoking were eliminated. The prevalence of smoking in the nationally representative NOWAC study was assessed and multivariable‐adjusted HRs were used as valid estimates of relative risks. The formula described in the WHO global report 8, was used to compute PAFs:

$$\text{PAF}\;=\;\frac{Pe\times (RRe-1)}{Pe\times RRe+(1-Pe)},$$

where the notation *Pe* is the proportion of persons in the population exposed to the risk factor, that is, ever smokers and *RRe* is the relative risk in the exposed compared to unexposed group; that is, ever smokers compared with never smokers in the final multivariable proportional hazards regression model, including all previously listed covariates. We calculated the two‐sided 95% CI's for the PAFs using the PUNAF Stata module 9. The analyses were performed with SAS‐version 9.4 (SAS Institute, Cary, NC, USA)/STATA‐version 13.1 (Stata Corp, College Statistics, TX, USA).

## Results

During >2 million person‐years with an average follow‐up time of 13.2 years, a total of 915 EOC cases (667 [73%] invasive and 248 [27%] borderline) were identified. The cases were classified as either serous (*n* = 554, [61%]), mucinous (*n* = 126, [14%]), endometrioid (*n* = 59, [6%]), or others (*n* = 176, [19%]). Table S1 shows that 30.8% of women were current, 34.5% former, and 18.0% passive smokers and the remaining 16.7% were never smokers.

Table 1 shows that compared with never smokers, current smokers had a significantly increased risk of borderline tumors of 69% (HR = 1.69 [95% CI 1.10–2.61]). The corresponding PAF attributed to current smoking was 17.2% (95% CI 3.8–28.7). The HR and PAF estimations in overall mucinous tumors when current smokers were compared to never were HR = 1.67 (95% CI 0.96–2.96), and PAF = 16.5% (95% CI −1.8–31.5). Current smokers who had smoked 20 or more years also had increased HR estimates compared with never smokers for the five outcomes, of which EOC overall (HR = 1.29 [95% CI 1.03–1.62], *P*_trend_ = 0.02), and borderline (HR = 1.85 [95% CI 1.16–2.95], *P*_trend_ = 0.01) tumors were significant (Table 1).

**Table 1:** Multivariable^a^ hazard ratios (95% confidence interval) of epithelial ovarian cancer overall by invasiveness status and by histological subtypes according to various measures of smoking status at enrollment compared with never smokers in NOWAC study 1991–2012 (*N* = 153,234)

	EOC overall *N* = 95		Invasive^b^ *N* = 667		Borderline^b^ *N* = 248		Serous^c^ *N* = 554		Mucinous^c^ *N* = 126	
	Cases	HR 95% CI	Cases	HR 95% CI	Cases	HR 95% CI	Cases	HR 95% CI	Cases	HR 95% CI
Smoking status										
Never	150	1	122	1	28	1	91	1	16	1
Passive	158	0.98 (0.79–1.23)	116	0.91 (0.71–1.18)	42	1.29 (0.79–2.10)	98	1.00 (0.75–1.33)	23	1.28 (0.67–2.44)
Former	275	0.99 (0.81–1.21)	207	0.96 (0.81–1.21)	68	1.11 (0.70–1.76)	172	0.97 (0.75–1.26)	32	1.05 (0.56–1.96)
Current	332	1.16 (0.98–1.47)	222	1.03 (0.81–1.30)	110	1.69 (1.10–2.61)	193	1.10 (0.84–1.43)	55	1.67 (0.96–2.96)
Smoking duration (years)^e^										
Former										
0–19	196	1.04 (0.83–1.30)	149	1.02 (0.79–1.32)	47	1.11 (0.680–1.82)	122	1.04 (0.78–1.38)	21	0.98 (0.49–1.96)
20+	79	0.98 (0.73–1.32)	58	0.93 (0.67–1.30)	21	1.18 (0.64–2.18)	50	0.96 (0.66–1.40)	11	1.21 (0.52–2.79)
*P*_trend_ d		0.99		0.76		0.58		0.91		0.70
Current										
0–19	60	1.08 (0.78–1.48)	42	1.03 (0.71–1.50)	18	1.32 (0.71–2.49)	31	0.92 (0.60–1.40)	14	2.22 (1.03–4.76)
20+	272	1.29 (1.03–1.62)	180	1.16 (0.90–1.50)	92	1.85 (1.16–2.95)	162	1.24 (0.93–1.66)	41	1.76 (0.93–3.32)
*P*_trend_		0.02		0.25		0.01		0.11		0.13
Pack‐years of smoking^e^										
Former										
0–9	203	0.99 (0.80–1.24)	153	0.96 (0.75–1.24)	50	1.10 (0.68–1.79)	132	1.03 (0.78–1.36)	20	0.87 (0.43–1.73)
10+	72	1.14 (0.85–1.55)	54	1.13 (0.80–1.59)	18	1.23 (0.65–2.31)	40	0.98 (0.66–1.46)	12	1.71 (0.76–3.86)
*P*_trend_ d		0.47		0.63		0.52		0.98		0.28
Current										
0–9	111	1.17 (0.90–1.52)	75	1.06 (0.78–1.44)	36	1.64 (0.97–2.77)	58	1.00 (0.70–1.42)	25	2.25 (1.15–4.41)
10+	221	1.29 (1.02–1.63)	147	1.18 (0.90–1.54)	74	1.78 (1.10–2.89)	135	1.28 (0.94–1.72)	30	1.60 (0.82–3.12)
*P*_trend_ d		0.03		0.23		0.03		0.09		0.28
Age at smoking initiation^e^										
Former										
20+	81	0.84 (0.63–1.10)	63	0.82 (0.60–1.12)	18	0.90 (0.49–1.65)	42	0.70 (0.48–1.01)	12	1.20 (0.55–2.60)
0–19	189	1.15 (0.91–1.46)	139	1.11 (0.85–1.45)	50	1.30 (0.79–2.16)	126	1.23 (0.91–1.66)	20	0.97 (0.48–2.00)
*P*_trend_ d		0.23		0.44		0.27		0.14		0.93
Current										
20+	109	1.18 (0.91–1.53)	82	1.16 (0.86–1.56)	27	1.35 (0.78–2.34)	52	0.96 (0.67–1.37)	22	2.01 (1.02–3.97)
0–19	217	1.28 (1.01–1.63)	137	1.12 (0.85–1.49)	80	1.87 (1.15–3.06)	139	1.33 (0.98–1.81)	30	1.49 (0.75–2.94)
*P*_trend_ d		0.05		0.42		0.01		0.05		0.34
Number of cigatettes/day^e^										
Former										
0–9	166	0.97 (0.77–1.22)	120	0.90 (0.69–1.18)	46	1.21 (0.74–1.98)	103	0.96 (0.71–1.29)	20	1.04 (0.52–2.08)
10+	95	1.11 (0.84–1.46)	73	1.12 (0.82–1.53)	22	1.09 (0.60–1.97)	61	1.12 (0.80–1.59)	10	1.04 (0.45–2.41)
*P*_trend_ d		0.52		0.58		0.74		0.56		0.92
Current										
0–9	167	1.31 (1.03–1.66)	119	1.26 (0.95–1.65)	48	1.60 (0.97–2.64)	94	1.22 (0.89–1.67)	24	1.60 (0.82–3.15)
10+	155	1.16 (0.90–1.50)	97	1.02 (0.76–1.37)	58	1.70 (1.03–2.82)	94	1.14 (0.82–1.58)	28	1.92 (0.97–3.80)
*P*_trend_ d		0.29		0.92		0.06		0.47		0.07

a Adjusted for age at menarche (≤12, >12 years), number of full‐term pregnancies (0, 1 or 2, 2+), age at first full‐term birth (nulliparous, ≤19, >19–24, and >24–29, ≥30 years), age at last birth (≤24, >24–29, >29–33, ≥34 years), infertility (yes, no, missing) menopausal status (postmenopausal pre‐ or perimenopausal, hysterectomy before 53 years, hormonal replacement therapy use before 53, missing), age at menopause (≤45, >45–50, >50–52, ≥53 years), educational attainment (≤19, >9–12, >12–16, ≥17 years, missing), physical activity score in the year preceding cohort enrolment (scored as 1–5 low to high level, missing), alcohol intake (teetotalers, ≤4, >5–9, and, ≥10 g/day, missing), BMI (missing, ≤18.49, >18.49–24.9, >24.9–29.9, and, ≥30 kg/m^2^), oral contraceptive use (yes/no, missing), duration of oral contraceptive use (≤3, >3–7, ≥8 years), hormonal replacement therapy (yes, no, missing), age at start using hormonal replacement therapy (≤45, >45–49, ≥50 years), history of breast cancer in mother (yes, no, missing).

b All histological subtypes.

c Include invasive and borderline tumors.

d Trend tests include never smokers.

e In the respective models additional missing in the main exposures qualifying smokers among ever smokers were excluded (age at start smoking *N*_missing_ = 1241, pack‐year *N*_missing_ = 19, average number of cigarettes smoked per day *N*_missing_ = 4418, duration of smoking in years *N*_missing_ = 19).

When current were compared with never smokers the HR estimates for endometrioid and “other” tumors were nonsignificantly decreased with 6% (HR = 0.94 [0.41–2.12]) and increased with 17% (HR = 1.17 [95% CI 0.75–1.85]), respectively.

Table 2 shows the serous and mucinous subtype categories stratified into invasive and borderline tumors: only the risk of borderline mucinous was significantly increased (HR_current vs never_ = 2.17 [95% CI 1.06–4.45]). The PAF attributed to current smoking was 24.7% (95% CI 3.8–28.7) for borderline mucinous tumors.

**Table 2:** Multivariable^a^ hazard ratios (95% confidence interval) of serous and mucinous epithelial ovarian cancer overall by invasive status according to various measures of smoking status at enrollment compared with never smokers in NOWAC study 1991–2012 (*N* = 153,234)

	Invasive tumors				Borderline tumors			
	Cases	Serous *N* = 397	Cases	Mucinous *N* = 43	Cases	Serous *N* = 157	Cases	Mucinous *N* = 83
Smoking exposure								
Never	72	1	7	1	19	1	9	1
Passive	72	0.96 (0.69–1.34)	8	1.01 (0.38–2.96)	26	1.17 (0.64–2.14)	15	1.49 (0.65–3.41)
Former	128	0.98 (0.74–1.32)	13	0.97 (0.36–2.60)	44	0.99 (0.56–1.75)	19	1.12 (0.50–2.52)
Current	125	0.99 (0.73–1.35)	15	1.02 (0.38–2.79)	68	1.46 (0.84–2.53)	40	2.17 (1.06–4.45)
Smoking duration (years)^c^^,^ d								
0–19	118	1.06 (078–1.44)	11	0.96 (0.36–2.55)	35	0.91 (0.51–1.62)	24	1.47 (0.67–3.27)
20+	135	1.04 (0.77–1.42)	17	1.12 (0.44–2.88)	77	1.59 (0.93–2.72)	35	1.97 (0.90–4.30)
*P*_trend_ b		0.82		0.75		0.02		0.07
Pack‐years of smoking^c^^,^ d								
0–9	138	1.04 (0.75–1.35)	16	1.08 (0.43–2.71)	52	1.11 (0.64–2.91)	29	1.48 (0.68–3.22)
10+	115	1.13 (0.82–1.56)	12	0.99 (0.37–2.69)	60	1.47 (0.85–2.56)	30	2.11 (0.95–4.69)
*P*_trend_ b		0.39		0.96		0.10		0.05
Age at smoking initiation^c^^,^ d								
20+	71	0.92 (0.58–1.15)	15	0.87 (0.26–1.88)	23	0.96 (0.45–1.58)	19	1.59 (0.73–3.49)
0–19	176	1.22 (0.90–1.64)	13	1.62 (0.64–4.11)	89	1.58 (0.93–2.71)	37	1.57 (0.69–3.55)
*P*_trend_ b		0.12		0.30		0.18		0.23
Number of cigatettes/day^c^^,^ d								
0–9	102	1.02 (0.76–1.39)	13	0.85 (0.33–2.20)	77	1.23 (0.72–2.11)	31	1.57 (0.72–3.40)
10+	139	1.07 (0.77–1.48)	13	1.31 (0.49–3.49)	58	1.36 (0.78–2.38)	25	1.67 (0.74–3.78)
*P*_trend_ b		0.68		0.48		0.29		0.26

a Adjusted for age at menarche (≤12, >12 years), number of full‐term pregnancies (0, 1 or 2, 3+), age at first full‐term birth (nulliparous, ≤19, >19–24, and >24–29, ≥30 years), age at last birth (≤24, >24–29, >29–33, ≥34 years), infertility (yes, no, missing) menopausal status (postmenopausal pre‐ or perimenopausal, hysterectomy before 53 years, hormonal replacement therapy use before 53, missing), age at menopause (≤45, >45–50, >50–52, ≥53 years), educational attainment (≤19, >9–12, >12–16, ≥17 years, missing), physical activity score (scored as 1–5 low to high level, missing), alcohol intake (teetotalers, ≤4, >5–9, and, ≥10 g/day, missing), BMI (≤18.49, >18.49–24.9, >24.9–29.9, ≥30 kg/m^2^, missing), oral contraceptive use (yes, no, missing), duration of oral contraceptive use (≤3, >3–7, ≥8 years), hormonal replacement therapy (yes/no, missing), age at start using hormonal replacement therapy (≤45, >45–49, ≥50 years).

b Trend tests include never smokers.

c In the respective models, additional missing in the main exposures qualifying smokers among ever smokers were excluded (age at start smoking *N*_missing_ = 1241, pack‐year *N*_missing_ = 19, average number of cigarettes smoked per day *N*_missing_ = 4418, duration of smoking in years *N*_missing_ = 19).

d Among ever smokers.

The multivariable‐adjusted HR's for current versus never smokers were significantly different between invasive and borderline tumors (*P*_heterogeneity_ = 0.04). None of the interactions tested between smoking status and alcohol consumption, educational attainment, BMI, menopausal status, and number of children were significant.

## Discussion

We found a statistically significant increased risk of borderline mucinous tumors in current smokers compared to never smokers. Current smoking was estimated to be responsible for one in four borderline mucinous tumors.

We calculated the PAFs and studied the effect of different measures of smoking exposures and found evidence of a dose‐response association between smoking duration and risks of overall EOC and risks of borderline tumors in current smokers.

Strengths of our study include the prospective and population‐based design, the large sample size, the long follow‐up time, the national population‐based registries, and detailed information on smoking history including passive smoking exposure. Moreover, the smoking exposure 10 and cancer incidence 5 are nationally representative, justifying the PAF estimation.

A limitation of our study is that updated information on smoking status was not considered in this analysis because that information was only available in a reduced simple size. In Norway, the proportion of daily smokers has decreased steadily from 36.5% in 1991 to 18.5% in 2012 11. In our study, the estimated prevalence of smoking was based on information collected between 1991 and 2007 (three waves of enrolment). Therefore, the decrease in smoking prevalence during this period is reflected in our data. However, as the majority of NOWAC women were recruited during the first wave of data collection (1991–1992), the decrease in smoking prevalence over time was only partially reflected in our PAFs estimates. This may result in overestimated PAFs of smoking. However, any possible misclassification in smoking status (current to former, former to current, never to current) would attenuate the displayed associations between current smoking and risk of EOC.

Another limitation of our study is the possible misclassification of histological types of EOC and invasiveness. We believe that a differential misclassification of EOC subtypes between current and never smokers is unlikely. In addition, we observe significant differences between invasive and borderline tumors, which is nonsupporting substantial misclassification in our data as this would have diluted the differences.

Borderline tumors are more common in younger women and have a much better prognosis than invasive tumors 12. The incidence rate of borderline tumors has increased in the last years in Nordic countries while the incidence of invasive ovarian carcinoma has decreased 13, 14. Hysterectomy with double adnexectomy is the recommended treatment in the presence of borderline tumors 15. This is considered invasive surgery, and for young women, this treatment has the serious consequence that they no longer can bear children.

With a larger number of mucinous cases, we found a similar risk and PAF estimation in mucinous tumors as a study from the European Prospective Investigation into Cancer and nutrition (EPIC) 2 that found 14% of mucinous ovarian cancer to be attributable to smoking. Unlike our cohort, the authors pointed out that EPIC is not a representative sample. To the best of our knowledge, the only other study reporting PAFs of overall ovarian cancer attributable to smoking 3, found this to be 2.6% of cases.

The interpretation of PAFs as the proportion of tumors that could be avoided if women did not smoke is justified when an established causal relationship exists as between smoking and mucinous tumors. We therefore also have estimated this PAF value although the corresponding HR estimate was not significantly increased in this study.

Our results are in agreement with respect to two recent meta‐analyses, the 51 epidemiological studies 16, and a recent pooled analysis of 21 case–control studies 17. As did we, the meta‐analysis of 51 epidemiological studies with >17,000 ovarian cancer cases found a more than double risk of mucinous borderline tumors for current compared with never smokers 16 and the pooled analysis of 21 case–control studies 17, found an odds ratio of 1.83 (95% CI 1.39–2.41).

In line with other previous studies 18, 19, we observed a borderline increased risk in mucinous tumors when comparing current to never smokers. This is explained both by an increased risk of borderline mucinous tumors and a nonassociation in invasive mucinous tumors. Although, with fewer mucinous cancer cases than in our present study, other studies have reported a statistically significant increased risks for mucinous tumors 16, 20, 21, 22. Nonetheless, when we stratify by invasiveness and histological subtype, a limitation is that we have few cases, especially for the mucinous tumors.

One plausible explanation for a stronger association between smoking and borderline tumors than invasive tumors is that somatic mutations in the KRAS gene are common in borderline tumors than in invasive, and are more frequent in mucinous compared to serous borderline ovarian tumors 23. Smoking‐induced KRAS mutations have been found in lung, pancreatic, and colon cancers 24, 25, 26, and a similar mechanism of oncogenesis might be applicable to borderline tumors.

In conclusion, among middle‐aged women, one in six mucinous tumors and one in four borderline mucinous tumors are attributable to smoking.

## Conflict of Interest

None declared.

## Supporting information


**Table S1.** Distribution of selected characteristics given as mean (SD) and percentages (%) according to smoking status, all at enrollment, Norwegian Women and Cancer Study 1991–2012, (*N* = 154,234).

## References

[1] Ferlay, J. , I. Soerjomataram , R. Dikshit , S. Eser , C. Mathers , M. Rebelo , et al. 2015 Cancer incidence and mortality worldwide: sources, methods and major patterns in GLOBOCAN 2012. Int. J. Cancer 136:E359–E386. 25220842 10.1002/ijc.29210

[2] Agudo, A. , C. Bonet , N. Travier , C. A. Gonzalez , P. Vineis , H. B. Bueno‐de‐Mesquita , et al. 2012 Impact of cigarette smoking on cancer risk in the European prospective investigation into cancer and nutrition study. J. Clin. Oncol. 30:4550–4557. 23169508 10.1200/JCO.2011.41.0183

[3] Parkin, D. M. , L. Boyd , and L. C. Walker . 2011 16. The fraction of cancer attributable to lifestyle and environmental factors in the UK in 2010. Br. J. Cancer 105(Suppl. 2):S77–S81. 22158327 10.1038/bjc.2011.489 PMC3252065

[4] IARC . 2012 Personal Habits and Indoor Combustions. Available at http://monographs.iarc.fr/ENG/Monographs/vol100E. PMC4781577 23193840

[5] Lund, E. , V. Dumeaux , T. Braaten , A. Hjartaker , D. Engeset , G. Skeie , et al. 2008 Cohort profile: the Norwegian Women and Cancer Study–NOWAC–Kvinner og kreft. Int. J. Epidemiol. 37:36–41. 17644530 10.1093/ije/dym137

[6] Lund, E. , M. Kumle , T. Braaten , A. Hjartaker , K. Bakken , E. Eggen , et al. 2003 External validity in a population‐based national prospective study–the Norwegian Women and Cancer Study (NOWAC). Cancer Causes Control 14:1001–1008. 14750540 10.1023/b:caco.0000007982.18311.2e

[7] Larsen, I. K. , M. Smastuen , T. B. Johannesen , F. Langmark , D. M. Parkin , F. Bray , et al. 2009 Data quality at the Cancer Registry of Norway: an overview of comparability, completeness, validity and timeliness. Eur. J. Cancer 45:1218–1231. 19091545 10.1016/j.ejca.2008.10.037

[8] World Health Organization . 2012 WHO global report on mortality attributable to tobacco. World Health Organization, Geneva.

[9] Newson, R . 2010 PUNAF: Stata module to compute population attributable fractions for cohort studies.Statistical Software Components S457193. Boston College Department of Economics, Boston, MA.

[10] Gram, I. T. , T. Braaten , E. Lund , L. Le Marchand , and E. Weiderpass . 2009 Cigarette smoking and risk of colorectal cancer among Norwegian women. Cancer Causes Control 20:895–903. 19274482 10.1007/s10552-009-9327-x PMC2694321

[11] Statistics Norway . 2015 Retrieve from http://www.ssb.no/en/helse/statistikker/royk.

[12] Kumpulainen, S. , T. Kuoppala , A. Leminen , M. Komulainen , U. Puistola , R. Sankila , et al. 2007 Surgical staging, treatment, and follow‐up of borderline tumors in different hospital categories: a prospective nationwide survey in Finland. Acta Obstet. Gynecol. Scand. 86:610–614. 17464592 10.1080/00016340701284707

[13] Bjorge, T. , A. Engeland , S. Hansen , and C. G. Trope . 1997 Trends in the incidence of ovarian cancer and borderline tumours in Norway, 1954–1993. Int. J. Cancer 71:780–786. 9180146 10.1002/(sici)1097-0215(19970529)71:5<780::aid-ijc15>3.0.co;2-c

[14] Skirnisdottir, I. , H. Garmo , E. Wilander , and L. Holmberg . 2008 Borderline ovarian tumors in Sweden 1960–2005: trends in incidence and age at diagnosis compared to ovarian cancer. Int. J. Cancer 123:1897–1901. 18661518 10.1002/ijc.23724

[15] Trillsch, F. , S. Mahner , L. Woelber , E. Vettorazzi , A. Reuss , N. Ewald‐Riegler , et al. 2014 Age‐dependent differences in borderline ovarian tumours (BOT) regarding clinical characteristics and outcome: results from a sub‐analysis of the Arbeitsgemeinschaft Gynaekologische Onkologie (AGO) ROBOT study. Ann. Oncol. 25:1320–1327. 24618151 10.1093/annonc/mdu119

[16] Beral, V. , K. Gaitskell , C. Hermon , K. Moser , G. Reeves , and R. Peto . 2012 Ovarian cancer and smoking: individual participant meta‐analysis including 28,114 women with ovarian cancer from 51 epidemiological studies. Lancet Oncol. 13:946–956. 22863523 10.1016/S1470-2045(12)70322-4 PMC3431503

[17] Faber, M. T. , S. K. Kjaer , C. Dehlendorff , J. Chang‐Claude , K. K. Andersen , E. Hogdall , et al. 2013 Cigarette smoking and risk of ovarian cancer: a pooled analysis of 21 case‐control studies. Cancer Causes Control 24:989–1004. 23456270 10.1007/s10552-013-0174-4 PMC3818570

[18] Gates, M. A. , B. A. Rosner , J. L. Hecht , and S. S. Tworoger . 2010 Risk factors for epithelial ovarian cancer by histologic subtype. Am. J. Epidemiol. 171:45–53. 19910378 10.1093/aje/kwp314 PMC2796984

[19] Gram, I. T. , T. Braaten , H. O. Adami , E. Lund , and E. Weiderpass . 2008 Cigarette smoking and risk of borderline and invasive epithelial ovarian cancer. Int. J. Cancer 122:647–652. 17918152 10.1002/ijc.23108

[20] Gram, I. T. , A. Lukanova , I. Brill , T. Braaten , E. Lund , E. Lundin , et al. 2012 Cigarette smoking and risk of histological subtypes of epithelial ovarian cancer in the EPIC cohort study. Int. J. Cancer 130:2204–2210. 21678398 10.1002/ijc.26235

[21] Terry, P. D. , A. B. Miller , J. G. Jones , and T. E. Rohan . 2003 Cigarette smoking and the risk of invasive epithelial ovarian cancer in a prospective cohort study. Eur. J. Cancer 39:1157–1164. 12736118 10.1016/s0959-8049(03)00195-3

[22] Tworoger, S. S. , D. M. Gertig , M. A. Gates , J. L. Hecht , and S. E. Hankinson . 2008 Caffeine, alcohol, smoking, and the risk of incident epithelial ovarian cancer. Cancer 112:1169–1177. 18213613 10.1002/cncr.23275

[23] Mayr, D. , A. Hirschmann , U. Lohrs , and J. Diebold . 2006 KRAS and BRAF mutations in ovarian tumors: a comprehensive study of invasive carcinomas, borderline tumors and extraovarian implants. Gynecol. Oncol. 103:883–887. 16806438 10.1016/j.ygyno.2006.05.029

[24] Baykara, O. , M. Tansarikaya , A. Demirkaya , K. Kaynak , S. Tanju , A. Toker , et al. 2013 Association of epidermal growth factor receptor and K‐Ras mutations with smoking history in non‐small cell lung cancer patients. Exp. Ther. Med. 5:495–498. 23403410 10.3892/etm.2012.829 PMC3570153

[25] Diergaarde, B. , A. Vrieling , A. A. van Kraats , G. N. van Muijen , F. J. Kok , and E. Kampman . 2003 Cigarette smoking and genetic alterations in sporadic colon carcinomas. Carcinogenesis 24:565–571. 12663519 10.1093/carcin/24.3.565

[26] Jiao, L. , J. Zhu , M. M. Hassan , D. B. Evans , J. L. Abbruzzese , and D. Li . 2007 K‐ras mutation and p16 and preproenkephalin promoter hypermethylation in plasma DNA of pancreatic cancer patients: in relation to cigarette smoking. Pancreas 34:55–62. 17198183 10.1097/01.mpa.0000246665.68869.d4 PMC1905887

