# Supplementary material for: Epithelial ovarian cancer subtypes attributable to smoking in the Norwegian Women and Cancer Study, 2012
Source: Cancer Med. 2016 Jan 14;5(4):720–7. doi: 10.1002/cam4.590 (PMC4831291; doi:10.1002/cam4.590)
Supplement: Supplementary file 1 — Table S1. Distribution of selected characteristics given as mean (SD) and percentages (%) according to smoking status, all at enrollment, Norwegian Women and Cancer Study 1991–2012, (N = 154,234). [file CAM4-5-720-s001.docx]

Table 1 SM: Distribution of selected characteristics given as mean (SD) and percentages (%) according to smoking status, all at enrollment, Norwegian Women and Cancer Study 1991–2012, (N = 154,234).

| Baseline charactersitics | **Smoking status** | | | |
| --- | --- | --- | --- | --- |
|  | **Never**  **N=25,771**  **(16,7%)** | **Passive**  **N=27,778**  **(18,0%)** | **Former N=53,191**  **(34,5%)** | **Current N=47,494**  **(30,8%)** |
| Age at enrolment (y) | 51.1 (8.7) | 49.4 (8.2) | 50.1 (7.9) | 47.8 (8.1) |
| Age at diagnosis (y)^1^ | 61.6 (9.0) | 59.2 (8.1) | 59.3 (8.2) | 58.6 (8.2) |
| Duration of follow-up (y) | 13.1 (5.9) | 13.5 (6.2) | 12.3 (6.1) | 14.0 (6.1) |
| Education (y) | 12.8 (3.8) | 13.0 (3.6) | 12.6 (3.6) | 11.4 (3.1) |
| BMI at enrolment | 24.4 (4.0) | 24.4 (4.0) | 24.6 (4.0) | 23.6 (3.8) |
| Mean phys. activity score | 5.7 (1.8) | 5.7 (1.8) | 5.8 (1.9) | 5.5 (2.0) |
| Height (cm) | 166 (5.8) | 166 (5.7) | 166 (5.6) | 166 (5.7) |
| Age at menarche (y) | 13.4 (1.4) | 13.3 (1.4) | 13.3 (1.4) | 13.3 (1.4) |
| Age at first birth (y)^2^ | 24.9 (4.4) | 24.6 (4.3) | 24.2 (4.6) | 22.9 (4.2) |
| Age at last birth (y)^2^ | 31.0 (4.9) | 30.2 (4.9) | 29.7 (5.1) | 28.3 (5.0) |
| Number of children | 2.4 (1.4) | 2.3 (1.2) | 2.2 (1.1) | 2.1 (1.2) |
| Number of children (%) |  |  |  |  |
| 0 | 11.5 | 9.4 | 8.7 | 9.5 |
| 1–2 | 44.5 | 51.9 | 55.9 | 56.6 |
| 3+ | 44.0 | 38.6 | 35.3 | 33.9 |
| Infertility (%) | 1.16 | 1.26 | 1.09 | 1.92 |
| Ever horm. contracept. use (%) | 57.3 | 47.2 | 39.0 | 38.1 |
| Duration of hormonal  contraceptive use (y)^3^ | 4.8 (5.1) | 5.0 (5.1) | 5.1 (5.2) | 5.1 (5.0) |
| Menopausal status at enrolment (%) |  |  |  |  |
| Premonopausal | 40.3 | 45.3 | 40.3 | 46.9 |
| Perimenopausal | 5.4 | 6.0 | 6.1 | 5.7 |
| Postmenopausal | 49.0 | 42.2 | 46.2 | 39.0 |
| Hysterectomy before age 53 | 1.9 | 2.4 | 2.4 | 2.7 |
| Hormone therapy use before age 53 | 1.5 | 1.8 | 2.6 | 2.8 |
| Missing | 2.0 | 2.2 | 2.4 | 2.8 |
| Age at menopause (y) (post menop)^4^ | 49.5 (4.3) | 49.3 (4.4) | 48.9 (4.4) | 47.7 (4.6) |
| Hormonal therapy use (%) |  |  |  |  |
| Never | 59.2 | 60.5 | 55.1 | 54.8 |
| Former | 11.2 | 10.9 | 14.7 | 10.7 |
| Current | 9.7 | 10.2 | 12.4 | 10.9 |
| Missing | 19.8 | 18.3 | 17.8 | 23.6 |
| Fam. history of breast canacer (%) | 5.4 | 5.5 | 5.3 | 5.0 |
| Teetotallers (%) | 38.1 | 26.9 | 16.0 | 18.5 |
| Alcohol consumption (g/day)^5^ | 3.5 (4.8) | 3.8 (3.9) | 4.7 (5.1) | 4.9 (6.8) |

^1^Age at overall cancer diagnosis i.e. concerning any cancer (not only EOC). ^2^For non-nulliparous women. ^3^Among hormonal contraceptive users. ^4^Age at menopause for postmenopausal women. Missing at age at menopause variable (N=20,781) was completed using the following rule: for women younger than 53 at enrollment, enrollment age was used as age at menopause otherwise for those older than 53, age at menopause was fixed at 53 years. ^5^Among alcohol consumers.
